# The Botanical Composition of the Diet of Grazing Cows During the Dry Season in a Subtropical Region of Mexico

**DOI:** 10.3390/ani16040612

**Published:** 2026-02-14

**Authors:** Felisa S. Jiménez-Peralta, Manuel Gonzalez-Ronquillo, Anastacio García-Martínez, Sherezada Esparza-Jiménez, Benito Albarrán-Portillo

**Affiliations:** 1 Centro Universitario UAEM Temascaltepec, Universidad Autónoma del Estado de México, Carretera Toluca-Tejupilco Km. 67.5, Temascaltepec 51300, Estado de México, Mexico; fjimenezp@uaemex.mx (F.S.J.-P.); agarciama@uaemex.mx (A.G.-M.); sesparzaj@uaemex.mx (S.E.-J.); 2 Facultad de Medicina Veterinaria y Zootecnia, Universidad Autónoma del Estado de México, El Cerrillo Piedras Blancas, Toluca 50295, Estado de México, Mexico; mrg@uaemex.mx

**Keywords:** alternative forages, drought, grazing, silvopastoral, botanical composition of the diet, selection index

## Abstract

The introduction of livestock into tropical and subtropical forest areas has displaced the original forest vegetation. Posterior surges of secondary vegetation have served as a source of alternative forage to cattle during scarcity periods. The objective of this study was to determine the botanical composition of cows’ diet (BCD) and the productive performance of lactating Brown Swiss cows during the dry season. The BCD was determined through microhistological analysis of faecal samples of grazing cows. Grazing areas were sampled to determine morphological and botanical composition (BCP). Forages were sampled to determine their chemical composition. Cows’ productive variables were recorded during March, April, and May of 2012. The BCP and BCD consisted of *Cynodon plectostachyus* and the woody species included *Vachellia farnesiana*, with smaller proportions of *Pithecellobium dulce*, *Guazuma ulmifolia*, and *Ficus* sp. These forages contributed 63, 48, and 47% of the dry matter, metabolizable energy, and metabolizable protein requirements of the cows. Therefore, it is concluded that alternative forages contributed significantly to addressing nutritional requirements during periods of pasture scarcity. Understanding the botanical composition of the diet of grazing cows allows for the development of management strategies based on the efficient use of local resources.

## Simple Summary

Tropical and subtropical cattle production systems use local resources to produce milk and beef under extensive grazing with limited supplementation during the dry season. Lactating cows graze in complex agroecosystems composed of native and introduced grasses in pastures with scattered shrubs and trees that are part of the cows’ diets, with varied nutritional composition. Our results provide information about the botanical composition of the cows’ diet and the nutrients obtained from forages like *Cynodon plectostachyus* and the woody species *Vachellia farnesiana*, *Pithecellobium dulce*, *Guazuma ulmifolia*, and *Ficus* sp. Knowledge of the alternative fodder resources consumed by the cows and their nutritional contributions can be used to develop efficient and sustainable management practices.

## 1. Introduction

In various regions of the world, there are forages grounded on animal production under extensive grazing and agrosilvopastoral systems [1], which are determined by the seasonality of forage production (rain and dry season). This seasonality determines the type of forage available (grasses, shrubs and trees) and its nutritional composition, which are factors that regulate cattle feeding behaviour and ultimately cattle productivity [2].

In tropical and subtropical regions of the Americas, the introduction of cattle has transformed forests by displacing native plant species with grass species established in monoculture pastures. Following the initial displacement of native species, secondary vegetation appears, which has been used by cattle during periods when forage is scarce in pastures or its nutritional quality decreases, representing a great diversity of alternative forage with a diverse nutritional composition [3].

The remaining original vegetation, as well as the secondary vegetation in tropical and subtropical areas where ruminants are reared, is widely diverse and complex, making it difficult to study; despite a number of animal production studies in the tropics [4], there is still a gap in knowledge about how the systems’ richness and species diversity, plants’ productivity and nutritional characteristics, animal preferences and foraging habits, and nutrients contribute to animals’ needs.

The above-mentioned gap has generated the need to study these types of systems, which are complex, with a great diversity of plant species and diverse and variable chemical composition depending on the season of the year. There are examples of the use of fodder trees and shrubs under modern intensive management systems in the tropics with reported animal production improvements [5,6]; however, there are still traditional animal production systems with minimal use of external inputs and based on local fodder resources that are little studied.

The southwestern region of the State of Mexico is a mountainous region with poor soils and steep slopes unsuitable for agriculture. Therefore, livestock farming is the best way to use land resources. Production units are called dual-purpose because they produce both milk and beef with low use of external inputs. During the rainy season, cattle feed exclusively on forages available in grazing areas. In this season, high temperatures and high precipitation result in forage overproduction, exceeding herd needs. Due to the geographic conditions, which do not allow the use of machinery to harvest and preserve the surplus forage, the best way to preserve forage in this type of production system is in situ, with the subsequent loss of nutritional value of available forages in pastures. As the dry season progresses, the quantity and quality of available forage decrease significantly, forcing producers to supplement their cattle to sustain milk production, maintain the body weight and body condition scores of lactating cows, and promote calves’ daily weight gain, which has a significant economic impact on production costs [7].

Producers know that livestock consume forage from trees and shrubs without knowing the nutritional characteristics of these alternative resources, so management strategies for better use of these resources cannot be developed [1].

The study hypothesis was that the botanical composition of the diet of lactating grazing cows includes fodder from woody species, to compensate for the lack of forage availability and diminished quality in the grazing areas. Therefore, the objective of this study was to determine the composition of the diet of lactating Brown Swiss cows in a dual-purpose production unit in the southwest of the State of Mexico, during the dry season (March, April and May).

## 2. Materials and Methods

### 2.1. Study Area

The study was conducted in a dual-purpose production unit in the municipality of Zacazonapan, State of Mexico, between 19°00′17″ and 19°16′17″ north latitude and between 100°12′55″ and 100°18′13″ west longitude, at an altitude of 1470 m above sea level. The climate is classified as semi-tropical, group A (Tropical Equatorial, hot, humid, no winter and temperatures above 18 °C), according to the Köppen climate classification, with summer rains and a dry season from November to May, classified as A (c, cool summer) (w2, dry winters), with an average annual temperature of 23 °C and an annual rainfall of 1115 mm [8]. The study was conducted during the months of March, April, and May of 2012, corresponding to the dry season. Each month was considered an experimental period (EP).

### 2.2. Production and Management of Experimental Cows

The dual-purpose production (PU) unit had an extension of 100 hectares of land with a perimeter fence and without paddock subdivision under extensive grazing management. Cows were free to choose where to graze within the PU extension, so the available herbage mass and its characteristics in the grazing areas varied considerably according to the area within the PU being grazed.

In total, 60% of the PU land was grazing area dedicated to forage production composed of native and introduced grasses, with scattered trees and shrubs (secondary vegetation), and 40% was dedicated to maize crop (*Zea mays*), which was subsequently used to prepare cows’ supplements.

Overall, management of the farm was minimal; there was no fertilisation or irrigation of the grazing areas, no conservation practices for surplus forage produced during the rainy season, and minimal inputs restricted to grains for cattle supplements during the dry season.

The PU herd consisted of 25 lactating Brown Swiss cows and the same number of calves. There were also nine dry cows and a sire, for a total of 60 animals. Male calves were sold at 6 months of age with a body weight range between 180 and 220 kg, whereas female calves were allocated to a separate PU, to be brought back to the main PU before first calving.

The PU infrastructure was limited to a basic milking parlour adjacent to a barn to store supplements. Milking was performed by hand once a day between 7:00 and 9:00 a.m., during which cows received 4.5 kg of dry matter (DM) per head/day of supplement and had access to water within the paddocks through a continuously flowing canal. The supplement consisted of a mixture of cracked corn ear with husk and soybean meal, containing 14% of crude protein.

Once milking ended and after all cows ate their supplements, the cows were released to start grazing. The main patterns of grazing activity were immediately after milking from 9:00 to 12:00 h and then from 15:00 to 18:00. Cows spent the nights on high ground within the grazing areas, and remained there 24 h, all year round.

### 2.3. Experimental Cows and Sampling

From the lactating cows in the herd, five cows were randomly selected to collect faecal samples taken directly from the rectum at the end of milking for two consecutive days in the last week of the EP, in order to determine the BCD, while the remaining lactating cows in the herd were monitored to record productive variables and collection of milk samples. The mean body weight (BW) of the cows in the herd was 400 ± 50 kg, with 3.6 ± 1.8 calvings, 115 ± 33 days of lactation, and a body condition score (BCS) of 2.5 points on a scale of 1 to 5 [9].

Milk yield (MY) was recorded for two consecutive days during the last week of each EP using a 20 kg capacity Rhyno^®^ electronic scale México City, México. Cows were weighed after milking on a 1500 kg capacity Smart Scale 200 (Gallagher^®^, Hamilton, New Zealand). Body condition score was determined at the same time as cow BW.

Milk components, fat, protein and lactose (g/kg), were determined in situ immediately after each cow was milked by analysing a sample (30 mL) with a portable milk analyser (Lactoscan Milk Analyzer^®^, Milkotronic, Nova Zagora, Bulgaria). Individual cow subsamples of milk were taken and transported to the laboratory to determine milk urea nitrogen (MUN) using enzymatic colorimetry [10].

### 2.4. Available Herbage Mass and Botanical Composition of the Grazing Areas

After milking, cows were followed to the grazing areas, and while the cows were grazing, six quadrats of 0.25 m^2^ were placed in a patch of pastures with similar characteristics to the area being grazed by the cows to obtain representative samples, in order to assess the availability of herbage mass (HM) (kg/ha). Herbage inside the quadrats was cut at ground level with shears, putting the material into plastic bags and then in a cooling box. Subsequently, in the laboratory, samples were weighed and then processed to determine the botanical composition, green and dead material, and leaf and stem composition of the grazing areas (BCP) according to [11]. The results represent the availability of herbage mass of pastures in the areas where cows were grazing during the sampling period at each EP and are expressed on a dry matter basis (kg of DM/ha).

#### 2.4.1. Woody Species

For two consecutive days during the last week of each EP, by direct observation during grazing after morning milking, and throughout the day until evening (6:00 p.m.), five experimental cows were monitored individually within the GA to observe the consumption of forage species at ten-minute intervals per grazing event [12]. Samples of plant species (leaves and stems) were taken by hand directly from the plant. The forage samples (approximately 200 g fresh base) were packaged fresh and transported to the laboratory for chemical analysis and to be processed for the microhistological assessment.

#### 2.4.2. Botanical Composition of the Diet

The BCD (percentage of species present) was estimated by identifying epidermal fragments in the faeces of the five cows using microhistological analysis. To ensure that the BCD analysis was representative, faecal samples were collected on the same days as the BCP was assessed. The faecal samples were dried in a forced-air oven at 70 °C for 48 h and then ground in a Willey mill with a 1 mm mesh.

Two sets of slides for the microhistological technique were prepared to determine herbage epidermic fragments. Permanent slides were prepared using material collected from the species consumed by the five cows randomly selected in each EP during grazing activity, and temporary slides were prepared using material from the faecal samples of the cows selected in each EP. The species that remained within the fields of the slides were counted to obtain the BCD. Fourteen slides were prepared per sample, per period of animal faeces collection, in which 280 fields were evaluated using a 10× optical microscope. Plant epidermic fragment prevalence in the slide field was assumed to be equivalent to prevalence in faecal samples and in grazed diets on a dry matter basis. In each field, the relative frequency (Fr), relative density (Dr), and selection rate (TS) or preference index (PI) were determined. The preference index is a ratio of the vegetation that makes up the diet to the vegetation present in the pasture. This indicator ranges from −1 to +1, with negative values for rejected components and positive values for preferred components, according to the procedures described by [13,14].

#### 2.4.3. Chemical Composition of Feed and Secondary Compounds

Samples of supplements and forages were taken on two consecutive days during the sampling period of every EP and dried at 55 °C to constant weight to determine DM. Feed samples were also taken. The chemical composition of the sampled fodders was analysed to determine dry matter (DM), crude protein (CP), NDF, ADF, and ADL according to [15] (AOAC 1995), and the in vitro digestibility of dry and organic matter was analysed according to [16]. The in vitro dry organic matter digestibility (IVOMD) of the main forage species consumed was estimated based on the results of the microhistological analysis. The Estimated Metabolizable Energy (ME) was derived from IVOMD [17]: eME (MJ/kg DM) = 0.01557 × IVOMD.

Secondary compounds, including total phenols, saponins and aqueous fraction, were determined in each of the woody species identified in the diet of the cows. Ten millimetres of extracts was fractionated by funnel separation with a double volume of ethyl acetate (99.7/100 analytical grade Fermont^®^, Monterrey, Mexico) to determine total phenols, by drying and quantifying the total phenols layer in the funnel. Subsequently, a double volume of n-butanol (99.9/100, analytical grade Fermont^®^, Monterrey, Mexico), was added to fractionate saponins [18]. The residual solution was considered the aqueous fractions (lectins, polypeptides and starch) [19].

### 2.5. Nutrient Intake Estimates

The dry matter intake (DMI) of individual lactating cows in the experimental herd (n = 25) was estimated indirectly from animal performance, using the nutritional composition of the forage and supplements, BW, BCS, and MY and composition, based on estimates from NASEM-Dairy-8 [20]. The programme was set using Brow Swiss as breed, characteristics of individual lactating cows in the herd, and indicating that cows grazed on pastures of moderate topography, and 23 °C of mean temperature.

From the dietary nutritional composition, the contributions of DMI (kg DM/day), metabolizable protein (MP) (g/day), and ME (MJ/kg) from supplements and forages to the cows’ requirements were estimated. Based on this, the contributions of these nutrients to the cows’ needs were determined.

The NASEM-Dairy-8 [20] uses Equation (1) to estimate dry matter intake as follows:

DMI (kg/day) = [3.7 + (parity × 5.7) + 0.305 × MilkE (MCal/day) + 0.022 × BW (kg) + (−0.689 − 1.87 × parity) × BCS] × [1 − (0.212 + parity × 0.136) × e^(−0.053×DIM)^] (1)

where parity is an adjustment factor ranging from 0 (all primiparous) to 1 (all multiparous) and BCS is scaled from 1 (thin) to 5 (obese). MilkE = milk net energy.

The metabolizable energy of feeds was estimated based on the digestibility of the organic matter using the following equation [21]:

ME (MJ/kg/DM) = 0.0157 × DOMD
      DOMD (g/kg) = OMD × (1000 − Ashes)/1000
(2)

### 2.6. Statistical Analysis

Data on HM, BCP, and BCD were analysed using descriptive statistics with the SAS PROC MEANS procedure of SAS^®^ OnDemand for Academics 2025 [22]. Animal productive variables were analysed using a mixed model, with the cow as a random effect and the experimental period as a fixed effect, using the SAS MIXED procedure [22] with the following model:

Y*_ij_* = μ + EP*_i_* + C*_j_* + e*_ij_*

where Y*_ij_* is the response variable, μ is the least square mean, EP*_i_* is the fixed effect of the experimental period (*i* = March, April and May), C*_j_* is the random effect of the cow (*j* = 1… 25), and e*_ij_* is the random error term.

## 3. Results

### 3.1. Herbage Mass

In Table 1, the available HM can be observed in the grazing areas. The average HM was 1932.7 (kg of DM/ha). In EP2, the HM estimated was almost twice as high as EP 1 and 3. This can be explained by differences in herbage availability in the areas where cows were grazing during the sampling period. As mentioned in the methodology, the extension of the grazing areas was around 60 ha, where cows were free to choose where to graze, since there were no subdivisions.

The green-to-dead material proportion was 50:50. Green material was considered if there was less than 50% of brown colour of the plant (stem or leaf), whereas dead material was considered if brown colour represented 50% or more of the plant. Of the available HM, 44% was leaf and 56% was stem. The average height in the grazing areas was 2.9 cm.

### 3.2. Botanical Composition

Table 2 shows the BCP and the CBD of the cows. The following grasses were identified in the grazing areas, *Cynodon plectostachyus*, *Paspalum notatum*, *Paspalum convexum*, *Eleusine indica* and *Urochloa brizantha*. *C. plectostachyus* was the most prevalent grass, in both the pasture, for an average of 57.5%, and in the cows’ diet, at 60.3%.

Additionally, Huizache (*Vachellia farnesiana*, formerly *Acacia farnesiana*) was found in the grazing areas, representing 36.1% of the pasture and 34.3% of the botanical composition of the cows’ diet. Other species of the Magnoliopsida class, like *Pithecellobium dulce*, *Guazuma ulmifolia*, and *Ficus* sp., were found in grazing areas, as well as in the BCD, which together represented 6.4% of the grazing areas and 5.4% of the cows’ BCD.

### 3.3. Chemical Composition of Forages

Table 3 shows the chemical composition of the main species identified in the diets of the cows. *C. plectostachyus* had a low nutritional value with 77.2 (g/kg of DM) of CP, low NDF, high levels of ADF and low ADL, resulting in a low in vitro organic matter digestibility (IVOMD) of 657.3 (g/kg) and an ME content of 9.6 (MJ/kg of DM). In contrast, the woody species had a high CP content, ranging from 228.2 of *V. farnesiana* to 109.5 (g/kg of DM) of *Ficus* sp.

Neutral detergent fibre content ranged from 346.8 (g/kg of DM) for *Ficus* sp. to 491.0 (g/kg of DM) for *G. ulmifolia*. However, the digestibility of woody species was lower than that of *C. plectostachyus*, with the highest value for *G. ulmifolia* at 651.2 (g/kg of DM) and the lowest for *V. farnesiana* at 470.7 (g/kg of DM). Metabolizable energy of consumed fodder species was generally low, ranging from 6.8 (MJ/kg of DM) of *F.* sp. to 9.6 (MJ/kg of DM) of *C. plectostachyus*, whereas the supplement ME was 12.0 MJ/kg of DM.

### 3.4. Content of Secondary Compounds

Table 4 shows the total phenolic (TP), saponin (SA), and aqueous fraction (AF) contents of the forage species identified in the cows’ BCD. *P. dulce* had the highest TP content, while the values for the remaining species were similar, ranging from 12.7 (g/kg of DM) of *C. plectostachyus* to 19.3 (g/kg of DM) of *Ficus* sp. Regarding SA, the highest values were for *Ficus* sp., with 37.4 (g/kg of DM), followed by *V. farnesiana* with 21.0 (g/kg of DM); the values for the remaining species ranged from 12.3 (g/kg of DM) to 14.1 (g/kg of DM) of *C. plectostachyus* and *G. ulmifolia*, respectively. Finally, the FA values ranged from 149.5 as the highest for *G. ulmifolia* to 90.3 (g/kg of DM) as the lowest for *C. plectostachyus*.

### 3.5. Productive Variables

Table 5 shows the productive variables of the cows. There were no significant differences in most of the cows’ productive variables (*p* > 0.05), except for milk fat and protein content (g/kg) and BCS (*p* < 0.01). Increases in milk fat content were observed from EP1 (32.0 g/kg) to EP3 (39.1 g/kg), while the opposite occurred in protein content, which decreased from 30.3 (g/kg) in EP1 to 29.7 (g/kg) in EP3. BCS increased significantly (*p* = 0.03) from 2.49 in EP1 to 2.52 in EP3.

The mean DMI was 11.4 (kg/cow/day), while the mean MY was 5.6 (kg/cow/day). The mean values for lactose, MUN, and BW were 42.5 (mg/kg), 14.0 (mg/dL), and 414.5 (kg/cow), respectively.

### 3.6. Nutrient Contribution

In Table 6, the contributions of dry matter, ME, and MP of supplement and forage (*C. plectostachyus*, *V. farnesiana*, *P. dulce*, *G. ulmifolia* and *F.* sp.) are shown. The supplement provided 37% of the estimated dry matter intake, while forages provided 63%. Regarding ME and MP contributions, the supplements provided 52 and 53%, respectively, while the forages provided 48 and 47%, respectively.

## 4. Discussion

### 4.1. Grazing Areas

The grazing conditions during the study were difficult for the cows, with important variation in grazing areas regarding forage availability and forage quality. In general, low availability and low pasture heights were factors that limited cows from higher dry matter intakes, and perhaps these temporary restrictions determined the selection of other alternative forages like woody species.

Low-input systems like the PU in this study rely on local forage resources that are produced at minimum cost, since no fertilisation, irrigation or forage preservation practices are performed. Therefore, despite the low productivity of the cows, milk and weaned calves generate sufficient incomes to cover the operation cost of the production unit and sustain the family economic needs of the farmer.

The live-to-dead and leaf-to-stem ratios were similar to those reported by [23] for pastures dominated by *C. plectostachyus* grass in the same study region during the same months as the present study, where the proportion of green and dead herbage was 23:77, while the leaf and stem proportion was 25:75, characteristic of pastures in tropical and subtropical conditions during the dry season. Tropical grasses like *C. plectostachyus* are of stoloniferous growth, which gives a higher proportion of stem over leaf, as in the present study [24]. In contrast to the findings of the present study, ref. [25] reported a composition in *C. plectostachyus* pastures associated with *L. leucocephala* cv. Cunningham under humid tropical conditions in southeastern Mexico, using two cutting interval schemes of 30 and 50 days. The leaf-to-stem ratio was 80:20, while the live-to-dead herbage ratio was 90:10. This result represents the potential of this type of pasture under intensive management.

Regarding nutritional composition, the values are within the range reported for pastures dominated by *C. plectostachyus* during the same study period [2]. However, the nutritional quality was lower than the values reported [25]. The cited work was carried out in a sub-humid tropical zone in association with *L. leucocephala*.

### 4.2. Forage Intake and Diet Composition

Animals exert preferences over the composition of their diet; what they decide to eat depends on the diversity of available forage and the frequency with which certain plants such as trees and shrubs are present, as well as their morphology and nutritional composition [2]. The microhistological technique has the limitation that the differential digestion rates can overestimate some species and, on the contrary, underestimate species sensitive to epidermal destruction during digestive track passage. Legumes and herbaceous plants tend to be overestimated and grasses tend to be underestimated [26]. In the present study, *C. plectostachyus* was the main grass in pastures and the BCD of the cows. For most of the study period (EP 1 and 2), cows grazed pastures of low-quality forage characterised by higher proportions of dead material. Only in EP3, where there was some precipitation, was the green material proportion higher than the dead material, implying forage in early growing stages low in NDF, representing a possibility of grass intake underestimation using the microhistological technique.

The estimated DMI of the average herd cows was 11.4 (kg of DM/cow), of which 4.5 kg was from supplement and 7.0 kg was from estimated forage consumed while grazing. Of the estimated total forage consumed, *C. plectostachyus* accounted for 60%, equivalent to 4.2 (kg of DM/day), *V. farnesiana* accounted for 34% or 2.4 (kg of DM/day), and *P. dulce*, *G. ulmifolia* and *Ficus* sp. together contributed 420 (g of DM/day), representing 6% of the total DMI. This is consistent with the BCP.

Overall, the amount of tree forage consumed was 2.8 (kg DM/day), which was higher than the 1.4 (kg DM/day) reported by [27]. The study by the cited authors was conducted in the same production unit three years after the present study, and throughout the whole dry season (December to May), unlike the present study (March to May). In the study mentioned, the woody species identified as components of the cows’ diet were *V. farnesiana*, *C. alata*, *P. dulce*, and *C. pentandra*, which represented 15.8% of the cows’ total DMI, which is lower than the 25% tree intake estimated in this study.

Studies have reported that the maximum inclusion level of woody forages without affecting the digestibility of the total diet, as well as the intake and productive performance of animals, is 30% of the total diet [3,18]. In the present study, in which the cows were free to choose the type of forage consumed, forage from woody species did not exceed this percentage.

In a study [3] with adult cows grazing in a tropical forest known as Acahual, it was reported that the cows’ diet during the dry season consisted of 26 fodder species, with a greater diversity of species consumed compared to the rainy season. However, the nutritional quality of the diet during the rainy season was higher in terms of CP, ADF, lignin, and saponins, while during the dry season, the diet had a higher content of NDF, as well as condensed tannins.

As the availability and quality of forage in pastures decreased, particularly that of *C. plectostachyus*, as indicated by preference indices, an increase in the preference index for species of the Magnoliopsida class (*P. dulce*, *G. ulmifolia* and *F.* sp.) was observed, although these represented a low percentage of the cows’ total DMI. Woody and shrubby plants are sources of protein and energy that also provide other nutrients such as vitamins and minerals during periods of scarcity of conventional forages. In addition, they contain secondary compounds, which improve protein utilisation and increase animal productivity, health, and overall animal welfare [28].

In addition to the BCP, the low consumption of species of the Magnoliopsida class may be related to a higher concentration of total phenols, saponins, and aqueous fractions compared to *V. farnesiana*, which could explain the greater consumption of this plant. However, it is also possible that the consumption of various woody plants is a strategy by the animal to minimise the risk of discomfort caused by consuming this type of forage, as well as to minimise the energy cost of detoxification when consuming forages with secondary compounds [28].

In this regard, the average CP of *V. farnesiana* (228.2 g/kg of DM) was higher than that reported by [29] (159.7 g/kg of DM). Meanwhile, the values for secondary compounds were within the normal range, making this woody species a suitable forage in terms of digestibility and CP content in the diet of cows [29,30].

### 4.3. Productive Variables of Cows

The mean MY was 5.6 (kg/cow/day), which is lower than the 6.8 (kg/cow/day) reported by [27]; the cited study was conducted in the same production unit. These differences may be related to the lower mean number of calvings (3.6 ± 1.8) and the average BW (414 kg) of cows compared to those used in the above-mentioned study, which reported 4 ± 1.2 calvings and an average BW of 460 (kg/cow).

Milk yields were higher than the 4.3 (kg/cow/day) reported by [31] with Zebu cows of different crosses (Brahman, Gyr, and Guzerat). However, they were lower than the 13.5 and 14.5 (kg/cow/day) reported by [32] in Holstein × Brown Swiss × Zebu cross cows grazing in a silvopastoral system with *L. leucocephala* and irrigated *Cynodon nlemfuensis* pastures in southeastern Mexico.

Fat content increased significantly as EPs progressed, which could be a result of the presence of rainfall in EP3 that could be related to the increased proportion of green material and better fibre quality, where the highest concentrations of milk fat were recorded. Although, on average, fat concentration was lower than reports in the literature for the Brown Swiss cows, these levels are within the range reported for cows in the region and the same PU [33]. Low milk fat concentration could be related to the low fibre quality, as it is known that tropical grasses are of low quality, containing high NDF fractions and low digestibility, as in the present study [34].

Conversely, the concentration of protein in milk decreased significantly. In this regard, it has been reported that heat stress can possibly cause a general decrease in the productive performance of cows and, in particular, protein content in milk [35].

The significance of BCS throughout the EPs is difficult to explain, since the changes were minimal, yet statistical significance was detected. Body condition scores were the same in EP 1 and 2, and then a significant increment was detected in EP3. This increment could be explained by two factors. A decrease in milk yields could be a result of the advance in lactation (mean of 205 days in milk); therefore, more nutrients were used to replenish BCS and BW. It could also be due to early rains that resulted in green forage in pasture, although the grazing condition remained difficult, as given by the low herbage availability and grazing height.

The supplementation also could have influenced the increments in BSC. Supplementation practices result in different types of response in cows. Responses in the short term included increments in milk and milk components, while medium- and long-term effects included improvements in energy balance, positively impacting fertility, as well as body weight and body condition score in the long term [36].

### 4.4. Contribution of the Diet to the Nutrient Requirements of the Cows

According to dry matter estimations, the forages combined (pasture and woody forages) contributed 63, 48 and 47% of the cows’ dry matter, metabolizable energy, and metabolizable protein requirements during the study period. The contribution of nutrients was lower than that estimated for the same production unit during the dry season by [28], who determined that the forages (pasture and trees) contributed 69, 63 and 60 of the cows’ DMI, ME and MP requirements.

## 5. Conclusions

Based on the microhistological technique used in the cows’ faeces analysis, the botanical composition of the diet during the dry season was composed of the grass *C. plectostachyus*, followed by woody species like *V. farnesiana* and, to a lesser proportion, *Pithecellobium dulce*, *Guazuma ulmifolia* and *Ficus* sp. Estimations suggest that the forage component of the cows’ diets represented 63% of dry matter intake, 48% of metabolizable energy and 47% of metabolizable protein intakes for their nutritional requirements, implying that forage component (pasture and woody species) contributes close to 50% or more with nutrients to address the cows’ needs during the dry season.

## 6. Study Limitations

The dry matter intake of the cows was estimated, not measured, so the estimations of grass and woody dry matter intakes, as well as their nutrient contributions to the nutrient needs of the cows, should be taken cautiously. Also, there is the possibility of over- and underestimation of some forages using the microhistological technique, due to differential digestion rates that can lead to overestimations or underestimations of species sensitive to epidermal destruction throughout the digestive track passage.

## Figures and Tables

**Table 1:** Availability of herbage mass and morphological composition of grazing areas (kg of DM/ha) per experimental period (EP) during the dry season.

Item	EP1	%	EP2	%	EP3	%	Mean	%	S.D.
Herbage mass	1434.0		2848.0		1516.0		1932.7		1061
Green herbage	682.5	48	1080.5	38	989.7	65	917.6	50	499
Dead herbage	751.5	52	1767.5	62	526.4	35	1015.1	50	690
Leaf	755.6	53	936.1	33	717.4	47	803.0	44	122
Stem	678.5	47	1911.9	67	786.6	53	1125.6	56	960

EP1 = March, EP2 = April and EP3 = May. S.D. = Standard deviation.

**Table 2:** Botanical composition of the grazing areas (BCP) and botanical composition of the diet (BCD) of lactating cows grazing during the dry season.

Item	Species	EP1		EP2		EP3	
		BCP	BCD	BCP	BCD	BCP	BCD
Grasses	*Cynodon plectostachyus*	59.5	62.8	57.1	60.4	56.0	57.8
Woody	*Vachellia farnesiana*	34.0	33.0	36.0	33.4	38.2	36.5
Other species	*Pithecellobium dulce, Guazuma ulmifolia*, and *Ficus* sp.	6.5	4.2	6.9	6.2	5.8	5.7

EP1 = March; EP2 = April; EP3 = May.

**Table 3:** Chemical composition of supplement (g/kg of DM) and forage species consumed by lactating cows grazing during the dry season.

Item	Supplement	*Cynodon plectostachyus*	*Vachellia farnesiana*	*Pithecellobium dulce*	*Guazuma ulmifolia*	*Ficus* sp.
DM	943.2 ± 50.4	714.1 ± 0.6	897.5 ± 0.1	607.4 ± 0.7	456.7 ± 0.4	702.4 ± 0.6
OM	846.0 ± 54.6	927.7 ± 43.9	946.2 ± 0.1	893.8 ± 13.8	883.9 ± 0.1	911.3 ± 7.4
CP	110.7 ± 4.6	77.2 ± 2.3	228.2 ± 0.4	195.6 ± 4.0	168.2 ± 0.4	109.5 ± 0.6
NDF	349.5 ± 1.2	637.0 ± 1.9	359.9 ± 4.0	362.2 ± 1.1	491.0 ± 0.9	346.7 ± 1.5
ADF	109.6 ± 1.3	374.2 ± 1.8	224.7 ± 1.9	227.4 ± 0.1	180.0 ± 1.7	203.2 ± 0.7
ADL	13.0 ± 0.3	42.0 ± 0.5	114.1 ± 1.6	104.9 ± 0.1	79.6 ± 0.3	79.8 ± 2.0
IVOMD	908.0 ± 0.8	657.3 ± 0.5	470.7 ± 2.8	512.6 ± 1.3	651.2 ± 4.8	473.0 ± 4.4
ME	12.0 ± 0.11	9.6 ± 0.7	7.0 ± 0.1	7.2 ± 0.1	9.0 ± 0.5	6.8 ± 0.1

DM = Dry Matter, OM = Organic Matter, CP = Crude Protein, NDF = Neutral Detergent Fibre, ADF = Acid Detergent Fibre, ADL = Acid Detergent Lignin. IVOMD = In vitro organic matter digestibility, ME = Metabolizable Energy (ME MJ/kg of DM).

**Table 4:** Secondary compounds content (g/kg of DM) (mean ± standard deviation) of species consumed by lactating cows grazing during the dry season.

Species	Total Phenols	Saponins	Aqueous Fractions
*Cynodon plectostachyus*	12.7 ± 2.9	13.8 ± 1.5	90.3 ± 28.8
*Vachellia farnesiana*	15.3 ± 0.1	12.3 ± 1.6	111.5 ± 10.3
*Pithecellobium dulce*	51.1 ± 0.4	21.0 ± 3.7	102.0 ± 21.3
*Guazuma ulmifolia*	14.0 ± 0.2	14.1 ± 2.3	149.5 ± 0.9
*Ficus* sp.	19.3 ± 0.1	37.4 ± 0.6	116.9 ± 4.3

Aqueous fraction = lectins, polypeptides and starch.

**Table 5:** Productive variables per experimental period (EP) of lactating cows grazing during the dry season.

Item	EP1	EP2	EP3	*p* =	SEM
DMI (kg/cow/day)	11.5	11.3	11.5	0.45	0.40
Milk (kg/cow/day)	5.9	5.6	5.3	0.15	0.27
Fat (g/kg)	32.0 ^a^	35.1 ^ab^	39.1 ^b^	0.001	1.98
Protein (g/kg)	30.3 ^a^	30.4 ^a^	29.7 ^b^	0.004	0.31
Lactose (g/kg)	41.6	43.3	42.5	0.63	1.1
MUN (mg/dL)	13.8	14.4	13.7	0.92	2.0
BW (kg)	420.6	406.3	416.7	0.16	11.9
BCS	2.49 ^ab^	2.42 ^a^	2.52 ^b^	0.03	0.04

DMI = Dry matter intake, MUN = Milk urea nitrogen, BW = Body weight, BCS = Body condition score. Different superscript within the same row indicate significantly differences.

**Table 6:** Estimated contribution of dry matter intake (DMI) (kg/DM/day), metabolizable energy (ME) (MJ/day) and metabolizable protein (MP) (g/day) of feeds to the nutrition requirements of lactating cows grazing during the dry season.

	DMI	ME	MP
Supplement	4.5 (37%)	61.9 (52%)	462.0 (53%)
Forage *	7.0 (63%)	57.6 (48%)	403.3 (47%)
Total	11.5	119.5	865.3

* *C. plectostachyus* (60%), *V. farnesiana* (34%), and *P. dulce*, *G. ulmifolia*, and *Ficus* sp. (6%). Estimations from NASEM-Dairy-8.

## Data Availability

The raw data supporting the conclusions of this article will be made available by the authors on request.
